# Supplementary material for: Are carbon emissions trading and green financial instruments synergistic? -Comprehensive quantitative research based on content analysis
Source: PLoS One. 2024 Mar 7;19(3):e0298601. doi: 10.1371/journal.pone.0298601 (PMC10919601; doi:10.1371/journal.pone.0298601)
Supplement: S1 Data — (ZIP) [file pone.0298601.s001.zip › Data/地级市Z_F工作报告.zip!/╔╜╢1⁄2/╬1⁄2╖╗╩╨/└┤╫╘╣1⁄2╓┌║┼í╛┬φ┐╦╩2╛▌═°í┐_2015.docx]

各位代表:

现在,我代表市人民政府向大会报告工作,请予审议,并请市政协委员和其他列席会议的同志提出意见。

2014年工作回顾

在省委、省政府和中共潍坊市委的正确领导下,在市人大及其常委会、市政协及社会各界的监督支持下,市政府与全市人民一道,开拓创新,扎实奋斗,圆满完成了市十六届人大三次会议确定的目标任务。

经济保持平稳健康发展。全市实现地区生产总值4786.7亿元,增长9.1%。完成一般公共预算收入430.2亿元,增长12.1%。完成固定资产投资3969.1亿元,增长15.7%。实现社会消费品零售总额1979.5亿元,增长12.6%。居民消费价格总水平上涨1.3%。科学应对我市有水文记录以来最严重的旱情,保证了用水安全,夺取了粮食丰收。

经济结构调整扎实推进。加快构建“1669”现代产业体系,对重点产业进行专业诊断咨询,制定完善产业发展规划,分行业建立产业基金,突出培育骨干龙头企业和重点园区,促进产业集群发展。农业加快转调创。粮食总产达到93亿斤,肉蛋奶、蔬菜、水产品产量稳中有升,农村改革扎实推进,国家蔬菜种业基地建设取得重要成果。全市新增各类现代农业园区110个、农民合作社4292家、家庭农场2080家。“三品一标”认证数量达到1608个。工业加快转型升级。全市完成工业技改投资1550亿元,高新技术产业、新兴产业占比分别提高1.6个和2.1个百分点,规模以上工业利税、利润分别增长10.1%和9.2%。市内新增科技孵化器20万平方米、省级以上高新技术企业123家。实施国家、省重大科技项目122项,获得省以上科技进步奖8项,发明专利授权增长52.8%。福田汽车研究院、通信技术研究院等开工建设 侯卫东官场笔记。潍柴林德液压国产化、福田汽车一工厂二期及中高档卡车扩能技改工程、盛瑞8AT产业化等重大项目顺利实施。国家生物基材料产业化集群获批建设。规模以上工业企业核心业务信息化覆盖面达到25%。淘汰落后产能任务全面完成。服务业加速发展。已建在建各类总部经济园区38个,新引进企事业单位总部(分支机构)410家。新增各类金融主体87家,全市金融存贷款余额分别达到5536.2亿元、4450.5亿元。电子商务交易额达到1300亿元、增长60.5%。文化创意产业增加值突破200亿元。5A级物流企业、五星级国际酒店实现零的突破。服务业增加值达到1898.5亿元,占比提高1.5个百分点。

区域发展布局更加优化。突破滨海保持强劲势头。率先推行“三张清单”“三制三零三全”管理模式,新增配套园区24.5平方公里,新开工过亿元项目108个,国家住宅产业化基地、通用航空产业园成功落户,14个县市区工业园一期配套基本完成,滨海区各项主要经济指标增幅保持20%以上。9个生态小镇规划编制完成。科教创新园区5所院校实现招生,在校师生达到3万人。山东(潍坊)公共实训基地加快建设。提升市区扎实推进。各专项规划基本编制完成,市区财政、规划、城管、环保等管理体制改革已经到位。“13364”各项任务全面展开,落实重点项目161个。市区老旧小区改造、中小学幼儿园建设计划启动实施,开工建设中小学校81所,竣工45所。5个老旧小区改造提升工程惠及群众5000户。重点片区(园区)加快建设,中国食品谷完成投资15.1亿元,食品总部基地等重点项目加快建设运营,一批新的重点项目签约入驻。潍坊总部基地完成规划设计和工商注册。测绘地理信息产业基地“一中心、两园区”基本建成。两河开发顺利启动。总体规划和6个专项规划编制完成,“两河”防洪工程列入国家规划。开工建设产业项目344个。4个县市入围全国“百强县”,16个镇入选全国重点镇,建成新型农村社区147个。全市城镇化率达到53.6%,提高1.8个百分点。重大基础设施建设实现新突破。潍坊港2万吨级码头建成投用,万吨级集装箱船实现靠泊。鲁辽陆海货滚甩挂运输大通道成功首航。潍日高速滨海连接线开工建设,潍县路改造工程具备通车条件。济青高铁市域线路及站点优化对接。潍坊机场运送旅客超过39万人次,同比增长194%；货邮吞吐量突破2万吨,机场迁建新址确定。

改革开放深入推进。在全省率先建立政府部门权力清单和服务清单制度,市级权力事项由8523项压减到3123项。工商注册便利化改革成效显著,新登记市场主体、注册资本分别增长42%和110%。市级公共资源交易平台成功运行。农村土地承包经营权确权登记颁证、农村集体资产改制基本完成。齐鲁农村产权交易中心投入运营,东亚畜牧产品交易平台获准筹备。规模10亿元的市再担保公司注册运行,全市注册基金总规模达到115.6亿元。新增上市公司1家、“新三板”和齐鲁股权交易中心挂牌企业25家。完成进出口总额177.8亿美元,跃居全省第四位。实际到账外资9亿美元。我市成为国家境外活体畜牧产品进口重要口岸。市综合保税区加快复制上海自贸区政策,北区建设及跨境电子商务申报工作顺利开展。“走出去”取得新进展,境外研发机构达到22处,10家企业在境外建立能源资源基地。成功举办了鲁台会、风筝会和第四届中国画节等重要展会。

生态环境持续改善。坚持铁腕治污、刚性减排,加快建设生态美丽潍坊。“386”环保行动扎实推进。508个重点减排工程投入运行。关停环保不达标企业308家、生产线29条,新淘汰20吨以下燃煤锅炉66台,PM2.5浓度同比改善18.9%,“蓝天白云、繁星闪烁”天数达到204天,列全省第五位。主要污染物减排完成省定任务。全市公共自行车规模达到4.98万辆、年周转量6000万人次。“211绿色低碳建筑行动”开工项目45个,完成建筑面积450万平方米。城乡环卫一体化经验在全省推广。节地、节水、节能工作扎实开展,环境能源交易中心建成运营。国家森林城市创建工作全面启动。

民生和社会事业全面发展。25件民生实事全面完成,民生支出占一般公共预算支出的比重达到60.1%。推动创业带动就业。建成市级以上创业孵化基地18处、面积达到217万平方米。潍坊创业大学正式开学。全市发放创业小额担保贷款11.4亿元,落实贴息资金6000万元,带动就业3.7万人。城乡就业连续11年实现“双十万”。惠及472万居民的基本养老保险制度调整完善,城乡一体的居民医保制度全面建立,有量化标准的民生保障项目全部达到或高于省均水平。省定保障房建设任务提前完成。改造提升城乡中小学校舍164万平方米。基础教育综合改革经验在全省推广。基层中医药服务能力提升工程、“云医院”建设试点正式启动,心理健康服务体系率先建立。文化事业繁荣发展,创建为全国未成年人思想道德建设先进城市和全省首批公共文化服务示范区。“单独两孩”政策全面落实,人口自然增长率控制在6.37‰。安全生产、食品药品安全监管和金融风险防控体系更加健全。综治维稳和社会治安管理进一步加强,群众安全感测评继续保持全省前列。双拥共建、国防动员和民兵预备役建设经验被济南军区推广。对口支援、扶贫开发、民族宗教等工作取得新成绩。成功创建为全国文明城市,实现了潍坊多年的夙愿。

政府自身建设进一步加强。扎实开展党的群众路线教育实践活动,“四风”突出问题得到有效整治,各类会议、文件大幅减少,市直部门单位公用经费继续压缩。新一轮市、县政府机构改革顺利完成,政务信息公开、电子政务工作进一步加强。严格执行市人大及其常委会决议决定,自觉把民主政治协商纳入决策程序,主动听取社会各界意见。共办理人大代表建议151件、政协委员提案294件。推行政府重要文件、合同合法性、合理性审查,政府智库、法律顾问制度已经建立。党风廉政建设与反腐败工作主体责任和监督责任进一步落实,行政监察和审计监督进一步加强,全市纪检监察机关共立查案件2406件,给予党纪政纪处分2264人。

各位代表,在宏观经济下行、各种矛盾错综复杂的形势下,这些成绩来之不易。这是省委、省政府和中共潍坊市委坚强领导的结果,是市人大、市政协和社会各界同心同德、有效监督的结果,是全市上下、驻潍部队和武警官兵团结奋斗、开拓进取的结果,也是广大港澳台同胞、海外侨胞、国际友人、在外潍坊籍乡亲关心支持的结果。在此,谨代表市政府表示衷心感谢!

各位代表,我们也清醒地看到,全市经济社会发展仍然存在不少矛盾与问题。宏观经济下行压力加大,企业经营成本持续上升,部分企业经营困难。产业结构不合理,高端产业、高效益企业、高附加值产品占比不高。创新创业能力不够强,国际化水平不够高,经济发展的内生动力不足。财政收支矛盾突出,一些领域存在潜在风险。节能减排、环境保护压力大,安全生产基层基础仍然薄弱。城乡居民收入增长不够快,社会保障等基本公共服务供给水平还不够高。政府职能转变还没有到位,法治政府建设任务繁重。部分公务人员法治意识不强,转变作风成果还不牢固。个别部门、单位仍然存在设租寻租和利益输送行为,反腐败斗争形势仍然严峻。加快适应新常态成为摆在各级政府和工作人员面前的新课题。对这些问题,将采取措施,认真加以解决。

2015年工作总体要求和目标任务

今年是全面深化改革的关键之年,是全面推进依法治国的开局之年,也是全面完成“十二五”规划的收官之年。做好今年的政府工作,要全面贯彻党的十八大、十八届三中、四中全会精神和省委、省政府决策部署,以习近平总书记系列重要讲话精神为指引,按照“走在前列、起好带头作用”的总体要求和“四个深入推进”的指导意见,自觉在全国、全省大局中定位,主动适应新常态,坚持稳中求进工作总基调,以提高发展质量、效益为中心,统筹“四一三”战略重点和战略布局,加大转调力度,强化创新驱动,着力改革攻坚,加强民生保障,努力在新起点上开创各项工作新局面。

全市经济社会发展主要预期目标是:地区生产总值增长8.5%左右；一般公共预算收入增长9.5%左右；社会消费品零售总额增长12.5%左右；固定资产投资增长15%左右；外贸进出口总额增长6%左右；城乡居民人均可支配收入分别增长9%和10%左右；城镇登记失业率控制在3.8%以内；人口自然增长率控制在9.5‰以内；居民消费价格涨幅控制在3%左右。全面完成上级下达的节能减排任务。这是综合考虑各方面因素,结合潍坊实际提出的,需要经过艰苦努力才能完成。

各位代表,做好今年工作,必须把“走在前列、起好带头作用”作为各项工作的立足点；把调整结构作为稳中求进的平衡点；把推进产业升级作为创新驱动的着力点；把优化营商环境作为改革开放的突破点。既勇于应对挑战,又善于抢抓机遇；既直面困难,又坚定信心；既重法治、守规矩,又勇于创新、敢于担当,始终保持昂扬向上的精神状态,抢占新一轮发展制高点,努力实现更高层次新发展。重点做好以下工作:

(一)保持经济稳定增长。把稳增长作为今年工作的首要任务,强化投资、消费、出口均衡拉动,确保全市经济持续稳定增长。发挥投资的关键作用。认真贯彻积极的财政政策和稳健的货币政策,统筹调结构、稳增长,着力增加投量、优化投向、加快投速、强化投效。实施好“六大工程包”,重点抓好总投资900亿元的十大重点工程、总投资2000亿元的100个重点项目,确保完成固定资产投资4350亿元。引导资金重点投向社会民生、节能环保、现代产业等重点领域,推进内涵效益型投资增长。以法治理念、创新思维破解项目审批慢、落地难瓶颈,提升投资效能。广开融资渠道,用足用活中央、省、市等政府投资,引导扩大外来投资,拓展市场融资,鼓励企业增资,增加社会融资总规模。强化消费的基础作用。抢抓消费转型升级机遇,多措并举促进大众消费,打造多点支撑的消费增长格局。落实收入分配制度改革各项措施,完善最低工资调整机制,稳步提高社会保障水平,增加社会消费能力。实施健康养老、信息消费等“六大领域消费工程”,加快培育消费新热点。推进内贸转型升级,建好公益性农产品批发市场,打造中心城区16条特色商业街和23个便民市场,大力发展高端连锁经营,规划建设乡镇多功能商贸中心。实施信息基础设施提升计划,中心城区重点公共场所3年内实现免费无线信号全覆盖。实施商务诚信建设行动计划,加大消费维权力度。稳定扩大出口。实施境外拓展计划,巩固传统市场、开拓新兴市场,确保全市出口额增长5%左右 落霞小说。保持经济稳定增长,归根到底要保障企业健康运行。强化经济发展以企业为本的理念,既重视发挥市场在资源配置中的决定性作用,又注重更好发挥政府作用,共同促进企业健康发展。分层次建立企业需求动态数据库,密切关注企业整体运行状况,研究破解企业“三难三贵”等共性问题的具体措施,增强企业生存发展能力。对标国内一流和国际规则,实施“营商环境优化工程”,营造各类市场主体生生不息、竞相发展的良好环境。全面落实促进民营经济创新发展意见,推动民营经济大发展、大繁荣,为稳增长提供支撑。优化政策机制,促进“个转企、小升规、规改股、股上市”,推动各类市场主体上档升级。

(二)推进产业转型升级。深入实施农业转调创、传统产业改造、新兴产业培育、服务业提速“四项工程”,按照“腾笼换鸟、凤凰涅槃”要求,以提高人均劳动生产率和投入产出率为核心,继续邀请专业机构对重点产业进行诊断咨询,逐一“敲开核桃”,一行业一对策,在更高层次上明确发展方向、工作重点,加快把特色产业做亮、传统产业做强、新兴产业做大,构建现代产业体系。

发展现代农业。农业是百业之基,也是我市的优势产业。潍坊农业最有条件“走在前列、起好带头作用”。要紧紧围绕“三个导向”,用好国家现代农业综合改革试点、全国供销社综合改革试点、全国农业国际合作示范区三块“金字招牌”,深化改革,加快转调创,力争在全省、全国率先走出产出高效、产品安全、资源节约、环境友好的现代农业发展路子。毫不放松抓好粮食生产。加快构建现代农业生产经营体系,大力发展农业龙头企业,提升规模化经营、标准化生产、企业化运营、社会化服务水平。进一步完善农产品质量监管体系,更加严格管理投入品,推行强制性例行速检,加快建立全程可追溯、互联互享的食品安全信息平台,创建国家农产品质量安全区。实施“五位一体”品牌农业发展战略,打造农业品牌强市。强化科技、人才、金融支撑,争创国家级农业高新技术开发区和农村合作金融创新发展示范市。实施农业良种化工程、农业新技术推广计划,推广土壤改良等“六项”新技术。实施农业节水改造等水利工程,加快建设现代水利示范市。

做优传统产业。机械装备、汽车制造、石化盐化、纺织服装、食品加工、造纸包装是我市的传统优势产业,也是我市产业转型升级的基础产业。充分发挥市场的决定性作用,同时更好发挥政府作用,促进传统优势产业转型升级、做优做强。改造提升一批。实施“两化融合”计划,加快建设工业4.0产业园等产业转型平台,重点组织100家企业示范引领,1000家企业启动实施,建设一批智能工厂。实施“互联网+”行动计划,促进云计算、大数据、物联网等新技术新业态与产业深度融合,推动制造产业智能化、智能制造产业化。实施制造服务化推进计划,重点抓好100个示范项目、促进100家企业启动实施、100家企业编制完成计划、系统培训1000名企业主管。实施优势产业提升计划,在更宽视野寻标对标,一行业一对策,一企业一计划,重点实施好1000个技改项目,促进产品升级、技术工艺更新、营销模式创新、产业链做优做强。加快实施林德液压国产化、福田汽车一工厂二期、盛瑞8AT产业化、生物基材料集群化等重大项目,扶持、引进一批新型业态、重点企业、关键技术,推动主导产品向终高端、绿色低碳、智能、进口替代和品牌产品延伸。推动一批优势产业、一批优势企业和一批名牌产品,瞄准国际先进标准,打造一批有国际竞争力的产业基地、知名企业和国际品牌。优化重组一批。运用市场机制,引导企业重组、联合、参股、并购和国际合作,支持骨干龙头企业组建产业集团和产业联盟,加速产业链垂直整合、金融教育科技等资源优化配置。有序转移一批。支持过剩、低效产能向低成本地区有序转移；支持有市场前景、能达标排放的企业和产业在有条件的镇、村布局产业园。实施新一轮退城进园计划,加速中心城区及周边部分企业有序转移,转型升级。依法淘汰一批。落实国家、省下达淘汰落后、过剩产能任务。完善分区域逐步加严的产业、环境标准,倒逼淘汰一批“两高一低”产能,加速产业更新。

做大新兴产业。突出电子信息、海洋动力装备、节能环保、生物医药、智能装备、新能源汽车等重点产业,加快膨胀规模,增强竞争力。年内,新兴产业占比再提高2个百分点以上。强化龙头带动。培育引进、优化重组并重,加速行业龙头企业进入全省一流、全国先进行列。做特做强园区。按主导产业规划布局特色园区,促进产业聚集发展。重点抓好光电、生物医药、通用航空等10个新兴产业园区建设。鼓励龙头企业、各类基金领办创办特色产业园区。强化项目支撑。逐个产业集中谋划实施一批重大项目,集中抓好智能无线音响、工业机器人等127个总投资1020亿元的新兴产业项目。加大支持力度。全面落实各项政策,进一步充实现代产业基金规模,优先保障要素供给,加强与国内外知名企业和基金战略合作,新组建一批产业基金。突破关键技术。坚持行业主导、企业主体、政府扶持,突破一批行业关键技术。重点抓好可穿戴式电子产品、智能测控装置等关键技术。

做强服务业。抢抓社会消费转型升级、生产性服务业突破发展新机遇,推动服务业发展提速、层次提升、占比提高。突出发展现代服务业。坚持不懈抓好总部经济、创意设计、现代金融、信息消费、电子商务、休闲旅游等重点服务业,逐一完善规划,落实工作计划,突破关键环节,促进加速发展。拓展发展领域。坚持问题导向,抓住党委政府关注、人民群众期盼、社会有关方面期待的领域,加强引导,加大投入,加快培育产业。加快发展现代教育、医疗健康、养生养老等“十大民生产业”,大力发展个性、绿色、人文、便利化高端服务业。推进三次产业融合发展。出台促进生产性服务业发展意见,促进生产性服务业跨越发展。农业服务业,大力发展苗木繁育、种子研发等“十类”新型服务业。制造服务业,引导有条件的企业,设立专门机构,制订专项政策,吸纳专业人才,实施专项计划,发展个性化定制、整体解决方案、融资租赁、再制造等新兴服务业。推动经济文化融合发展,挖掘我市丰厚的文化遗产,推动文化资源产品化、文化产品创意化、创意产品产业化、文化产业集群化,打造新的产业亮点。重点规划建设好10个市级现代服务业集聚区、50个特色园区。

完善促进产业转型升级的体制机制。完善主体功能区域规划管理和产业标准管理,定期更新产业标准、产品目录和负面清单,形成分区域、分行业逐步加严的产业标准。大力发展资本市场,引导企业上市、挂牌及开展私募股权交易,依靠市场实现要素优化重组、上档升级。建立与结构调整相适应的要素保障、生态管理、政策支持、政务服务措施,激发企业转调内在动力。探索教育、人才、科技、金融与产业融合发展的体制机制,加快发展动力转型。实施标准品牌战略,推出一批潍坊标准和潍坊高端品牌。大力培育有世界眼光、现代意识、创业精神,敢担当、有办法的现代企业家队伍。

(三)大力实施创新创业战略。更大力度推进创新、鼓励创业,加快形成大众创业、万众创新的局面,打造国内一流的创新城市、创业城市。

强化创新驱动。坚持市场导向和产业化方向,坚持企业主体地位和市场决定性作用,推动以科技创新为核心的全面创新。加速成熟科技成果产业化步伐。对全市科技创新需求进行调查摸底,建立成熟科技成果项目库和重点项目会商推进机制,有限资源要素优先保障成熟科技成果产业化。建立重大科技成果转化联系人制度。深入推进科技合作。引导企业在全球范围内优化配置科技资源。加密组织企业与重点院所对接活动,探索建立产学研及各类基金常态化对接机制,深化与重点国家(地区)产学研合作。支持有条件的企业建设海外研发、孵化、技术转移中心。支持潍坊路演中心做大做强。规划建设好创新平台。支持市高新区加速对接国际规则,创新体制机制,打造全市创新高地。加快市科教创新园区建设步伐。促进清华、西安交大、中科院等技术转移中心运营,东北大学、山东理工大学、山东通信院、福田汽车等在潍研发机构加快建设,发挥好现有省级以上院士工作站等平台作用,再定向引进一批境内外知名院所或分支机构、发展一批新的合作平台。深化孵化器提升工程。开展孵化器转型试点。新发展科技孵化器10个以上、面积不低于30万平方米。加快建设市科技孵化中心。规划建设市科技型中小企业产业园和科技中介组织聚集区。强化企业主体作用。支持各类企业建立研发机构,加大科技投入,开展科技攻关。支持潍柴动力创建国家重点实验室、天瑞重工等企业建设省级示范工程技术研究中心。大力培育高新技术企业,加大对创新型小微企业扶持力度,高新技术产业占比再提高2个百分点左右。大力推动科技体制机制创新。深化科技成果产权制度、收益分配制度改革,让科研人员合法取得更多权益。更加严格保护知识产权,依法保护知识创造价值。加速科技成果市场化改革,高标准建设科技市场,让市场主体以投资的理念选择、评价、转化科技成果。大力发展风险投资等创新金融。市财政再安排2亿元充实市创新基金。

促进大众创业。把促进创业作为富民强市的战略举措,让一切创业的源泉充分涌流。加大政策支持。设立1亿元的创业带动就业扶持资金,年内小额担保贷款达到11亿元以上,带动就业3万人以上。培养中、高级创业培训师100人以上,培训各类创业人员2万人以上。拓展创业大学功能,探索建立“创业大学+教学点+实训基地”的创业培训模式。鼓励驻潍高校、职业学校实行“双训制”。搭建创业平台。鼓励支持各部门、驻潍各高校、境内外专业机构创办领办孵化器,鼓励支持存量房地产转办、合办孵化器。年内再新建成各类创业载体100个。建立孵化器认定、绩效管理和定期评估制度,探索推行孵化器公司化运营、专业化服务模式。优化创业环境。建立市创新创业促进联席会议制度,市政府每年至少专题研究一次创新创业工作,协调解决关键问题。设立服务“绿色通道”,完善项目库,开设服务网站、服务热线和新闻专栏。动员支持更多的在潍大学生和各类人才踊跃创新创业、更多的潍坊籍人才回乡创新创业、更多的海外人才来潍创新创业。设立“政府创新创业奖”,表彰一批“创新创业之星”。

加强人才队伍建设。更大力度实施人才强市战略。完善全市人才发展规划及行业、区域人才发展专项计划,统筹党政机关、经营管理、技术研发、中介服务等各类人才。鼓励各行业培育和引进行业领军人才(团队)。规划建设高端人才社区、专家公寓,配套建设国际学校,设立人才服务中心,为高端人才提供“保姆式”服务。创新人才评价、甄别、奖励机制,让有真才实学、突出贡献的人才得到丰厚回报,甚至“一朝即富”。

(四)深入推进重点领域改革。抓住影响全市科学发展的关键问题、制约转型升级的突出矛盾以及群众反映强烈的民生诉求,积极探索,破解难题。着力激发市场主体活力。落实已出台各项改革措施,继续深化行政审批制度改革,推进“减、压、转、放、免”,年内再下放一批市级行政审批事项。探索实行非生产类工程项目部分环节审批承诺制。推进商事制度改革。启动市级公共信用信息平台建设。深化国有独资和控股公司改革。继续推进民营企业公司化改造。完成市级生产经营类事业单位“事改企”。深入推进公共资源配置市场化改革。深化农村改革。扎实开展现代农业综合改革、供销合作社改革、农村合作金融改革试点。推动齐鲁农村产权交易中心规范化运行。探索建立财政转移支付、建设用地增加规模与农业转移人口市民化挂钩机制。进一步理顺收入分配关系。改革机关事业单位工资制度、养老保险制度。扎实推进新一轮财税体制改革,完善全口径政府预算体系,探索加强预算绩效管理的科学机制,全面推行预算公开。进一步完善政府各类引导基金规范管理运行办法。完成公务用车制度改革。深化投融资体制改革。减少和取消财政对竞争性行业的直接补助,大力推行政府与社会资本合作(PPP)等市场化支持方式。在公用事业领域推出一批项目向社会资本开放。全面落实“金创33条”,大力发展互联网金融,加快构建区域资本中心。引导支持企业加大直接融资比重,新增上市、挂牌企业15家以上,债券融资80亿元以上。加快建设“基金谷”。高标准规划建设文化艺术金融融合发展试验区。推进社会领域改革。深化中小学管理体制改革,开展学校自主办学试点。推动智慧教育发展。建设国家特殊教育改革实验区。制定深化医疗卫生改革指导意见,开展医疗卫生服务综合改革和“云医院”建设试点。探索成立医疗卫生服务集团,发展医疗联合体,健全促进分级诊疗政策机制。鼓励民营医疗机构加快发展和境外优质医疗机构合作办医,落实医师多点执业配套措施。

(五)全面提升开放发展水平。实施新一轮开放计划,综合运用国际国内两个市场、两种资源、两类规则,推动更高水平引进来,更大规模走出去。深度对接国家战略。主动融入国家“一带一路”、互联互通和中韩、中澳自贸区建设,办好中(日)韩产业博览会,规划建设好中外产业合作园区。依托东亚畜牧产品交易平台,规划建设国际畜牧产业园、中国—东盟国际食品贸易港。构建全方位开放格局。启动国际化五年计划,完善城市开放基础设施和双语标识,逐步建立与国际接轨的公共服务体系。促进各类开发区转型发展,依托市综合保税区加快复制上海自贸区制度创新成果,综合保税区北区年内达到验收条件。积极参与省电子口岸建设。加快推进外经贸“五个转变”。加快建设服务全市的跨境电商综合服务平台,扶持1000家企业向跨境电商延伸。依托骨干企业境外机构,设立潍坊境外产品营销中心,探索建设潍坊城。实施服务外包产业发展规划,争创国家服务外包示范城市。落实鼓励进口政策,扩大资源能源性产品、先进技术设备和关键零部件进口,支持增加原油、燃料油自营进口。支持企业开展跨国投资并购、资源能源开发、优势产能转移和国际研发合作。加快招商转型。着力推进环境招商、产业链定向招商、专业招商。分行业建立专业招商队伍,与境内外专业招商代理机构、基金公司开展战略合作,推进招商专业化；建立招才引智、招院引所、招商引资并重,产业招商与其他各领域招商并举的工作格局,促进全方位开放。制定重点招商目录和定向拜访计划,集中引进一批世界500强、大型央企民企、知名院所、领军人才(团队)、关键技术和行业龙头企业。认真抓好全国知名民营企业入鲁助推转调创等签约项目的落实。

(六)统筹城乡区域协调发展。深入实施重点区域发展战略,切实做好“整合、提升、创新”文章,提升区域统筹发展水平。突破滨海,着眼发展海洋经济、支撑和引领全市转型发展,统筹规划制定实施、统筹生态环保、统筹基础设施建设、统筹产业布局,加快建设港口经济区、海洋科技大学园、海洋动力机械装备产业园、绿色化工产业园、中外产业园、旅游度假区、中央商务区、羊口生物基新材料示范基地、下营科技兴海示范园等九个特色园区,在更高层次、更高水平上加快建设滨海海洋经济新区。突出抓好通用航空产业园、潍柴中速机、华创机器人、华春医药、石大昌盛等重点产业项目,加快建设未来大厦、在水一方、湖光山舍等城市配套项目。提升市区,以增强中心城区首位度为根本,按照“13364”整体布局,实施产业提升计划,拉开“两廊两核四区多园”产业发展格局,突出抓好光电产业园、中国食品谷、总部基地等重点片区(园区)开发建设。抓好一批房地产转型发展示范楼宇,引导房地产业转型发展。实施功能品质提升计划。以增强“六项功能”为目标,抓好中小学建设改造、“四通一治”、智慧潍坊建设等“七项工程”,让城市功能更完善、市民生活更便利。实施城市活力提升计划。继续深化城市管理体制改革,完善城市更新发展机制,集中布局创新创业资源,实施中心城市国际化示范工程,增强城市发展内生动力。深入开展“主题”活动,动员各级各部门、各行各业和广大市民共同参与、共建共享美好家园。实施城市品牌营销计划,构建立体营销网络,加大城市品牌宣传推介力度,提升城市美誉度。开发两河,加快河流水系生态建设,推动城镇化与农业现代化良性互动、经济发展与生态保护相得益彰。规划建设一批新型农村社区和特色园区,打造两河开发新亮点。鼓励城市资本参与城镇和新农村建设。深化扩权强镇改革,培育一批小城市,精心打造一批特色镇,全市城镇化率再提高2个百分点左右。加快重大基础设施建设。潍坊港实现3.5万吨级船舶通航。组建潍坊港集团,加快建设临港经济区。鲁辽陆海货滚甩挂运输大通道正式运营。机场实现临时对外开放,机场迁建完成预可研报告评审。潍日高速滨海连接线加快建设。开工建设疏港铁路。做好济青高铁和青银高速改扩建开工各项工作。启动城市地铁前期工作。加快推进“特高压入潍”工程。规划建设输气新管线、大型LNG调峰储配站等供气设施。完成引黄入白、引黄入峡调水工程,启动峡山水库增容工程。

(七)持续加大生态治理和保护力度。以对群众和子孙后代高度负责的精神,做好生态治理、环境保护各项工作,全面完成省政府下达的减排刚性目标和“386”环保行动预定目标,加快建设生态美丽潍坊。消灭污水直排。完善污水处理和管网配套建设,实现城区及重点镇区污水全部收集处理、城区再生水全部回用。实施河道治理和生态湿地建设工程。完成潍河、弥河等17条河流河道生态湿地修复、建设。减少大气扬尘。严控煤炭消耗总量,全社会煤炭消费总量控制在2010年水平,煤炭在一次能源中占比降到55%以内。拆除改造“禁燃区”内20吨及以下燃煤锅炉,进一步扩大市区“禁燃区”范围。整合供热市场,加大工业余热利用力度。全面落实城区扬尘污染防控主体责任,推进多领域气化改造。城市建成区外延5公里范围内预拌商品混凝土企业全部完成搬迁或密闭改造,确保颗粒物指标明显下降。整治空气异味。淘汰一批、搬迁一批、改造提升一批污染企业。全面治理挥发性有机物,深化废气综合整治,重点部位全部安装烟气自动在线监控设施。对各类超标排污行为实行“零容忍”。推行测土配方施肥和绿色控害技术,加强畜禽养殖污染治理,推动城乡环卫一体化镇村全覆盖,加大农业面源污染治理力度。开展城市能源管理。实施绿色建筑、可再生资源利用等9项工程,扎实推进中美低碳生态城市、国家循环经济示范城市试点。加快编制实施南部山区综合规划,建立南部水源涵养区、中部湿地保育区和北部平原水网生态区三大水生态功能区。扎实推进国家森林城市创建。完善集约节约用地机制。试行居民生活用水、产业用水阶梯价格制度,建立全社会节水体系。

(八)促进民生改善和各项社会事业发展。始终把保障和改善民生作为政府工作的出发点和落脚点,财政再紧张,也要谋划好事关群众长远利益的大事,解决好群众生产生活中的难事,办好群众日常生活离不开的小事,继续办好一批民生实事(附后),确保民生支出占比再提高1个百分点以上。大力促进就业。把促进就业摆到经济社会发展优先位置,制定就业促进政策,完善就业援助制度,设立就业特殊贡献奖。突出做好以高校毕业生为重点的青年和农民工就业,启动农民工“三项行动计划”,确保新增城镇就业、农村劳动力转移就业各10万人。提升保障水平。按照保基本、兜底线、促公平的要求,统筹财政、社会和居民个人,织密织牢民生保障网。加大扶贫开发力度,大力开展精准扶贫,减少贫困人口。研究综合措施,提升特困家庭保障水平。城乡居民基础养老金最低标准由每人每月75元提高到85元,医保财政补助标准由每人每年320元提高到380元。企业退休人员基本养老金再提高10%左右。低保重度残疾人救助标准由每人每月50元提高到80元。加强社会救助体系建设,进一步提高城乡低保、农村五保、临时救助标准和比例。新开工建设各类保障性住房3500套,改造棚户区1.4万套。探索将农民工和灵活就业人员纳入住房公积金制度覆盖范围。办好人民满意的教育。全市新改扩建中小学205所,加快解决好大班额问题；全市新改扩建标准化幼儿园100所。推广普及已有教育改革成果,及早解决师资力量不足、分布不均衡问题。大力发展民办教育,鼓励优质教育资源集团化办学、品牌化发展。支持各类社会主体参与、领办、合办职业教育；大力发展高等教育,支持市内各高校创新体制机制,开展国际国内合作；加大引入国内外知名高校、研究生院工作力度。加快发展公共医疗卫生、体育事业和健康产业。支持市内重点医疗机构改扩建,加大境内外优质医疗资源引进力度。完善社区卫生服务网络,构建“10分钟医疗服务圈”和“5分钟急救圈”。落实全市健康产业发展规划,打造健康产业集聚区。搞好养老服务业综合改革试点,加快社会化养老服务体系建设。建设全民健身服务体系,培育发展体育产业。办好“两项国际足球赛事”。繁荣发展文化事业和文化产业。经济社会越发展,文化的支撑引领作用越凸显。充分挖掘我市丰富文化资源,繁荣文化事业,做强文化产业。积极培育和践行社会主义核心价值观。深化潍坊历史文化研究,大力传承、弘扬潍坊优秀传统文化,培育新时代潍坊精神。深入实施文化惠民工程,加快建设现代公共文化服务体系。按照“立得住、传播得开”的要求,实施好文化精品工程、“潍水记忆”工程,打造“潍水文化”品牌。大力实施文化产业提升工程和重点项目带动战略,抓好192个重点文化产业项目,加大新型文化业态、文化品牌培育引进力度,提升世界风筝都、中国画都等文化品牌影响力。提升社会治理水平。全面落实市委推进依法治市实施意见,加快社会治理体系和治理能力现代化步伐。

实施全民普法依法治市规划,推动全社会学法尊法守法用法,崇尚法治、厉行法治。完善群众自我管理、自我教育、自我服务机制,全面强化基层民主自治。鼓励发展社会组织。加快建立统一的社会信用体系。依法全面落实安全生产监管责任、主体责任和各项工作措施,扎实推进安全生产标准化规范化建设,提升全社会安全发展能力。完善各类社会风险预警防范和处置机制,确保不发生区域性系统性风险。开展“食安潍坊”品牌引领和“399”专项整治行动,创建国家食品安全城市。扎实推进平安潍坊建设,加强社会治安综合治理,完善立体化治安防控体系。强化全社会防灾减灾意识,健全完善防灾减灾体系。深入开展双拥共建,加强国防动员和国防后备力量建设,推进军地融合发展。

同时,今年要集中全社会的智慧,精心编制好全市“十三五”发展规划。全面建设法治政府

出台法治政府建设规划纲要,将法治建设贯穿于经济社会发展全过程和政府建设各方面,加快建设职能科学、权责法定、执法严明、公开公正、廉洁高效、守法诚信的法治政府。

严格依法履行职能。政府各部门和广大公务人员要带头尊法学法守法用法,运用法治思维和法治方式推进工作。推行政府权力清单、责任清单、市场准入负面清单制度,明确政府权力边界。坚持法定职责必须为、法无授权不可为,对法定职责范围内的事情,要勇于负责、敢于担当,以踏石留印、抓铁有痕的精神抓好落实。对不作为、乱作为以及各种失职、渎职行为,要依法依规予以查究。加快建立行政权力运行、公开、监督、监察平台,让权力在阳光下运行。继续推进简政放权,全面推行行政审批规范化、便利化。建设投资项目在线审批监管平台,确保审批再提速20%以上。

健全依法决策机制。完善重大行政决策程序规则,严格执行公众参与、专家论证、风险评估、合法性审查、集体讨论决定等法定程序,建立重大决策第三方参与听证制度。完善政府智库、法律顾问参与决策机制,加快筹建市科学发展研究院。建立重大行政决策终身责任追究制和责任倒查机制。完善行政决策落实情况监督、评估制度。

深化行政执法体制改革。减少层次,提高效率,科学划分执法权限,合理配置执法力量。探索实行市、区一个层级执法。充实和加强县、镇执法力量。探索在县级政府设立统一的综合行政执法机构。建立行政执法机关与公检法机关信息共享、案情通报、案件移送制度,实现行政处罚和刑事司法无缝衔接。

严格公正文明执法。开展“行政执法规范年”活动。完善行政执法行为裁量标准和操作流程,严格执行执法全程记录、涉企检查网上报备制度。严格实行执法资格管理制度。严格落实行政执法责任制和责任追究机制 金庸小说。严厉查处重点执法领域违纪违法案件。

强化权力监督制约。认真落实市人大及其常委会决议决定,自觉接受人大依法监督和政协民主监督,主动听取民主党派、人民团体和社会各界意见建议,自觉接受司法监督、新闻舆论监督和社会公众监督。强化监察、审计监督和政府内部层级监督,健全行政执法监督机制。

加大反腐倡廉力度。严格落实党风廉政建设主体责任和监督责任,认真遵守政治纪律和政治规矩,深入贯彻中央八项规定精神和省、市具体规定,巩固、拓展群众路线教育实践活动成果,持之以恒反“四风”、改作风。进一步增强过“紧日子”思想,所有公务人员和一切公务活动都要厉行节约、杜绝浪费。市直部门公用经费预算再压减5%。坚持有腐必反、有贪必肃,坚决查处各种腐败行为和腐败分子。

各位代表,新常态,新挑战,新机遇。让我们紧密团结在以习近平同志为总书记的党中央周围,在省委、省政府和市委的坚强领导下,同心协力,改革创新,扎实奋斗,全面完成今年经济社会发展和“十二五”规划各项目标任务,为建设富裕文明新潍坊打下更加坚实的基础!
